# Targeting RET–interleukin-6 crosstalk to impair metastatic dissemination in breast cancer

**DOI:** 10.1186/bcr3608

**Published:** 2014-01-28

**Authors:** Andrea Morandi, Clare M Isacke

**Affiliations:** 1 Department of Experimental and Clinical Biomedical Sciences, University of Florence, Viale Morgagni, 50, I-51034, Florence, Italy; 2 Breakthrough Breast Cancer Research Centre, The Institute of Cancer Research, London SW3 6JB, UK

## Abstract

RET (rearranged during transfection) is a receptor tyrosine kinase overexpressed in a subset of oestrogen receptor (ER)-positive breast cancers whose expression is regulated by ER signalling. The article from the Hynes group has reported for the first time that RET expression can also be regulated by the inflammatory cytokine IL-6. Importantly, RET and IL-6 interact at a functional level to control migration and the metastatic potential of ER-positive breast cancer cells, in a process that is mediated by FAK activation. Further, targeting RET with receptor tyrosine kinase inhibitors was reported to be more effective than endocrine therapies in impairing metastatic dissemination *in vivo*, thereby indicating a level of RET regulation that is independent of ER.

## Background

Many breast cancers are characterised by amplification or overexpression of receptor tyrosine kinases such as ErbB2/human epidermal growth factor receptor 2 (HER2), epidermal growth factor receptor, insulin-like growth factor receptor and fibroblast growth factor receptor 1 that can drive tumour growth. Targeting ErbB2/HER2 with the specific antibody trastuzumab has changed the standard of treatment and has improved prognosis for ErbB2/HER2-positive breast cancer patients. However, as ErbB2/HER2-positive breast cancers represent approximately 25% of all breast cancer cases, there is a need to identify additional kinases that can acquire driver characteristics in breast cancer progression and impact on therapy response.

An increasing body of evidence has now documented that the receptor tyrosine kinase RET is overexpressed in a subset of oestrogen receptor (ER)-positive breast cancers that depend on oestrogens for their growth and survival [1-3]. Targeting ER function with endocrine therapy is the most common and effective treatment for this subset of breast cancers, but recent data have shown that RET signalling, driven by its ligand GDNF, has a negative impact on the response of ER-positive breast cancer cells to aromatase inhibitors [4] and tamoxifen [5,6].

## The article

**Figure 1:**
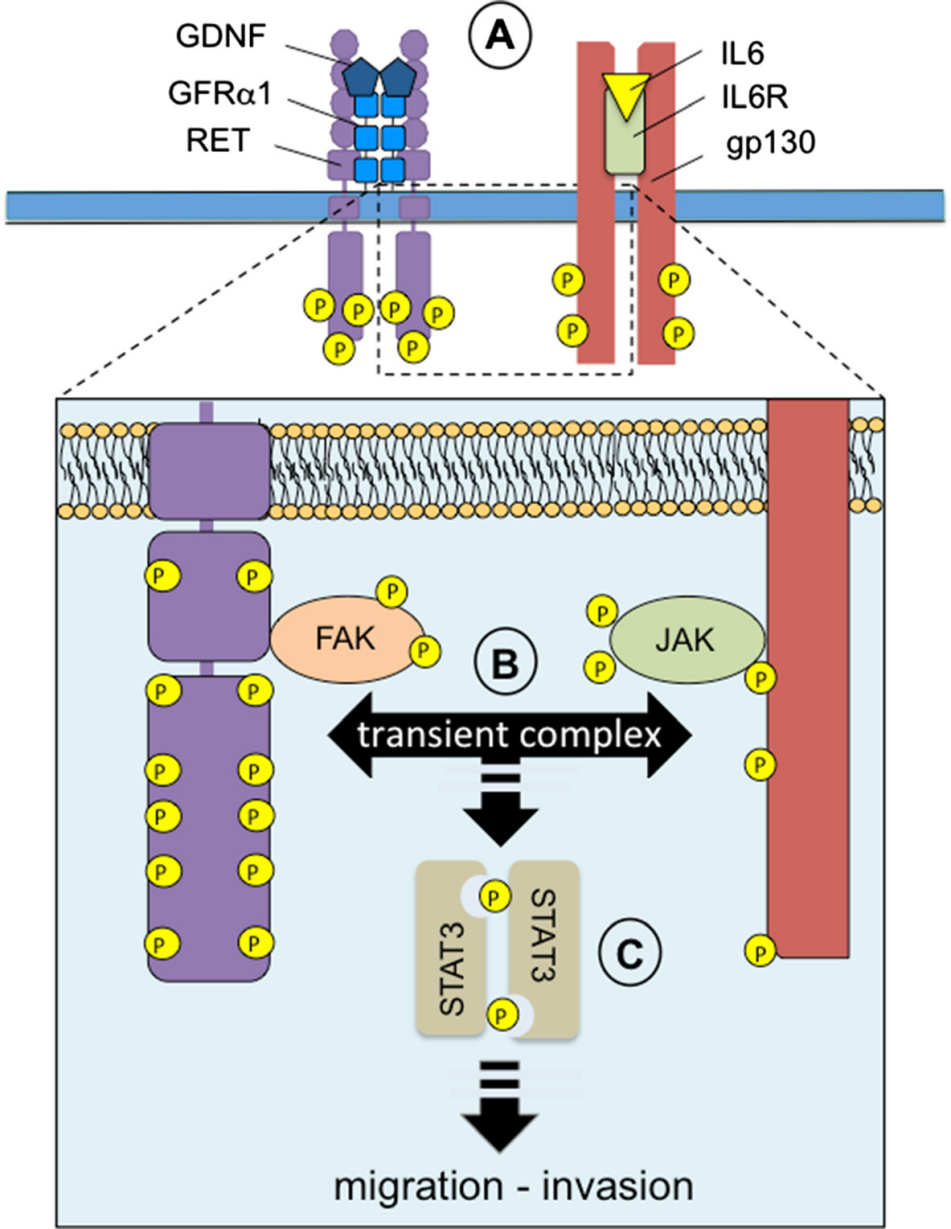
**RET–IL-6 interaction mediates breast cancer cell motility. (A)** GDNF binds to GFRα1 and induces RET activation. IL-6-mediated IL-6 receptor (IL6R) activation leads to co-receptor gp130 phosphorylation (P). **(B)** RET directly interacts with and activates FAK, while IL6R/gp130 activation induces JAK phosphorylation. Data presented by Gattelli and colleagues suggest a transient interaction between the IL6R:JAK and RET:FAK activated complexes that form in response to IL-6 and GDNF treatment, respectively [7]. **(C)** RET and FAK are essential to IL-6:JAK-mediated STAT3 activation underpinning the observed requirement for RET in IL-6-stimulated breast cancer cell migration and invasion.

The work recently published from the Hynes laboratory has uncovered a novel role for RET in migration and metastasis, in addition to confirming the relevance of RET in controlling proliferation and growth of ER-positive breast cancer [7]. Importantly, using *in vivo* models they demonstrate that tyrosine kinase inhibitors which target RET can block primary tumour growth of ER-positive breast cancers with an efficiency that is comparable with endocrine agents, the current standard of care for this subset of tumours. In contrast, the authors show that RET inhibition is more effective than endocrine agents (that is, fulvestrant and tamoxifen) in preventing metastatic dissemination. Using a multidisciplinary approach, they demonstrate that targeting RET and/or ER signalling impacts on the inflammatory response. In particular, they reveal a feed-forward RET–IL-6 loop in which RET activation increases IL-6 levels that in turn induce RET expression. Furthermore, IL-6 signalling can be blocked by RET inhibition and RET-mediated cell migration can be attenuated by an IL-6 blocking antibody. The functional link between RET and IL-6 comes from a reverse protein array analysis on xenograft samples, which revealed that RET inhibition results in phospho-FAK, phospho-STAT3 and phospho-AKT reduction. From this observation, FAK was demonstrated to act as intracellular integrator of RET–IL-6 signalling, with FAK activity being essential for both IL-6-mediated and RET-mediated cell migration (Figure 1).

## Viewpoint

The novel findings of this article are that RET inhibition impacts not only on primary tumour growth of ER-positive breast cancers but also on their metastatic dissemination, and that the promotion of migration and metastasis of ER-positive breast cancer cells promoted by IL-6 and RET signalling is mediated by FAK activity (Figure 1).

The implications of this study are multiple. Inflammatory response pathways were previously reported to be regulated by GDNF-mediated RET activation. Particularly, a GDNF–RET set of genes associated with poor prognosis and endocrine therapy resistance was largely populated by interferon-related genes [4]. Gattelli and colleagues for the first time highlight the functional interconnection between RET downstream signalling and inflammatory response in an endocrine therapy setting [7]. Moreover, although RET has been shown to be an ER-dependent gene [2], this current study additionally shows that IL-6 can induce RET expression. The importance of this observation is that fulvestrant administration, which degrades ER and thereby disrupts ER signalling, would be expected to negatively impact on RET expression. Instead, Gattelli and colleagues show that fulvestrant induces cancer cells to produce IL-6, resulting in increased RET expression and thus creating a feed-forward RET–IL-6 expression loop. This novel observation needs to be considered to completely understand the role of RET in breast cancer.

To date, most of the mechanistic insights into RET have been described in the ER-positive breast cancer subset. However, an aspect that may be underevaluated in Gattelli and colleagues’ article is that the clinical information is based on a cohort of patients enriched for ER-negative tumours (52/89, 58.4%). Particularly, approximately 70% of the triple-negative breast cancers analysed show a high RET expression. This observation raises the intriguing question of whether an inflammatory regulation of RET expression (for example, via IL-6 signalling) could drive ER-independent RET expression. In addition, the tumour microenvironment may contribute to potentiate RET activation and signalling. Proinflammatory cytokines were reported previously to induce the expression of the RET ligand GDNF [1]. In addition, cancer-associated fibroblasts mediate tumour-enhancing inflammation and produce IL-6 [8]. Consequently, given the current report that IL-6 promotes RET expression, the role of the GDNF–RET axis may be more relevant in those cancers characterised by an inflammatory response and an activated tumour microenvironment.

The second important finding of this current article is that RET and IL-6 are also connected at a functional level. Previous reports state that RET binds to the FERM domain of FAK, an interaction that results in transactivation of both proteins [9], and that RET is degraded by autophagy in cancer cells with altered/reduced FAK signalling, preventing RET binding to FAK at focal adhesions [10]. The novelty of Gattelli and colleagues’ article is that it demonstrates FAK is essential for IL-6-mediated RET-dependent cell migration. Both RET and FAK inhibition impaired IL-6-induced migration and metastatic ability of breast cancer cells, and, conversely, when FAK is inhibited, RET-induced and IL-6-induced migration is abolished.

These findings together have the intriguing therapeutic possibility of targeting FAK as a key signalling pathway downstream of RET to block tumour growth and metastatic potential not only in ER-positive breast cancers but also, potentially, in other breast cancer subtypes. This hypothesis may be an important strategy in tumours where an inflammatory response could increase the expression of the molecular players involved in RET–IL-6 crosstalk.

## Abbreviations

ER: Oestrogen receptor; FAK: Focal adhesion kinase; HER2: Human epidermal growth factor receptor 2; IL: Interleukin; RET: Rearranged during transfection.

## Competing interests

The authors declare that they have no competing interests.

## References

[B1] Esseghir S Todd SK Hunt T Poulsom R Plaza-Menacho I Reis-Filho JS Isacke CM A role for glial cell derived neurotrophic factor induced expression by inflammatory cytokines and RET/GFR alpha 1 receptor up-regulation in breast cancer Cancer Res 2007 67 11732 11741 10.1158/0008-5472.CAN-07-2343 18089803

[B2] Boulay A Breuleux M Stephan C Fux C Brisken C Fiche M Wartmann M Stumm M Lane HA Hynes NE The Ret receptor tyrosine kinase pathway functionally interacts with the ERα pathway in breast cancer Cancer Res 2008 68 3743 3751 10.1158/0008-5472.CAN-07-5100 18483257

[B3] Morandi A Plaza-Menacho I Isacke CM RET in breast cancer: functional and therapeutic implications Trends Mol Med 2011 17 149 157 10.1016/j.molmed.2010.12.007 21251878

[B4] Morandi A Martin LA Gao Q Pancholi S Mackay A Robertson D Zvelebil M Dowsett M Plaza-Menacho I Isacke CM GDNF-RET signaling in ER-positive breast cancers is a key determinant of response and resistance to aromatase inhibitors Cancer Res 2013 73 3787 3795 10.1158/0008-5472.CAN-12-4265 PMC3686594 23650283

[B5] Plaza-Menacho I Morandi A Robertson D Pancholi S Drury S Dowsett M Martin LA Isacke CM Targeting the receptor tyrosine kinase RET sensitizes breast cancer cells to tamoxifen treatment and reveals a role for RET in endocrine resistance Oncogene 2010 29 4648 4657 10.1038/onc.2010.209 20531297

[B6] Jan R Huang M Lewis-Wambi J Loss of pigment epithelium-derived factor: a novel mechanism for the development of endocrine resistance in breast cancer Breast Cancer Res 2012 14 R146 10.1186/bcr3356 23151593 PMC3906603

[B7] Gattelli A Nalvarte I Boulay A Roloff TC Schreiber M Carragher N Macleod KK Schlederer M Lienhard S Kenner L Torres-Arzayus MI Hynes NE Ret inhibition decreases growth and metastatic potential of estrogen receptor positive breast cancer cells EMBO Mol Med 2013 5 1335 1350 10.1002/emmm.201302625 23868506 PMC3799490

[B8] Erez N Truitt M Olson P Arron ST Hanahan D Cancer-associated fibroblasts are activated in incipient neoplasia to orchestrate tumor-promoting inflammation in an NFκB-dependent manner Cancer Cell 2010 17 135 147 10.1016/j.ccr.2009.12.041 20138012

[B9] Plaza-Menacho I Morandi A Mologni L Boender P Gambacorti-Passerini C Magee AI Hofstra RM Knowles P McDonald NQ Isacke CM Focal adhesion kinase (FAK) binds RET kinase via its FERM domain, priming a direct and reciprocal RET-FAK transactivation mechanism J Biol Chem 2011 286 17292 17302 10.1074/jbc.M110.168500 21454698 PMC3089571

[B10] Sandilands E Serrels B Wilkinson S Frame MC Src-dependent autophagic degradation of Ret in FAK-signalling-defective cancer cells EMBO Rep 2012 13 733 740 10.1038/embor.2012.92 22732841 PMC3410392

